# Virus-Like Particles Containing the Tetrameric Ectodomain of Influenza Matrix Protein 2 and Flagellin Induce Heterosubtypic Protection in Mice

**DOI:** 10.1155/2013/686549

**Published:** 2013-07-30

**Authors:** Li Wang, Ying-Chun Wang, Hao Feng, Tamanna Ahmed, Richard W. Compans, Bao-Zhong Wang

**Affiliations:** Department of Microbiology and Immunology and Emory Vaccine Center, Emory University School of Medicine, Atlanta, GA, USA

## Abstract

The ectodomain of matrix protein 2 (M2e) is highly conserved among influenza A viruses and can be a promising candidate antigen for a broadly cross-protective vaccine. In this study, a tetrameric M2e (tM2e) and a truncated form of flagellin (tFliC) were coincorporated into virus-like particles (VLPs) to enhance its immunogenicity. Our data showed that the majority of M2e in VLPs was presented as tetramers by introducing a foreign tetramerization motif GCN4. Intranasal immunization with tM2e VLPs significantly enhanced the levels of serum IgG and IgG subclasses compared to soluble M2e (sM2e) in mice. tM2e VLPs also induced higher M2e-specific T-cell and mucosal antibody responses, conferring complete protection against homologous influenza virus infection. The immunogenicity of tM2e VLPs was further enhanced by coincorporation of the membrane-anchored tFliC (tM2e chimeric VLPs) or coadministration with tFliC VLPs as a mixture, but not the soluble flagellin, inducing strong humoral and cellular immune responses conferring cross-protection against lethal challenge with heterotypic influenza viruses. These results support the development of tM2e chimeric VLPs as universal vaccines and warrant further investigation.

## 1. Introduction

Influenza A virus (IAV) is a negative sense single-stranded RNA virus responsible for annual seasonal epidemics worldwide and, occasionally, pandemics caused by emerging novel subtypes/strains derived by reassortment with avian or porcine viruses [1, 2]. Current influenza vaccines are based primarily on antibody responses against the hemagglutinin (HA) or neuraminidase (NA) and provide strain-specific protection only [3, 4]. Due to these limitations of current vaccines, it is crucial to establish a broadly cross-protective influenza vaccine, namely, universal vaccine. The appropriate presentation of an immunogen conserved in all influenza A viruses to the human immune system is important for an effective universal influenza vaccine.

The ectodomain of the influenza A M2 protein (M2e) is highly conserved among influenza A viruses and is considered to be a promising target for inducing cross-protection against different influenza A virus subtypes [5]. Some M2e-based vaccines protected mice from low-dose lethal virus challenge [6, 7]. However, in most studies, M2e was not presented in its native tetrameric form or its membrane-bound environment. Since antibodies specific to conformational epitopes presented in quaternary structures may be more effective at binding M2 on cell surfaces [8], a tetrameric conformation-stabilized recombinant M2e presented in a membrane-anchored form, such as those incorporated into VLPs, was predicted to be more immunogenic than other M2e forms.

Toll-like-receptor- (TLR-) based immune adjuvants can induce efficient mucosal adjuvant activity [9]. The bacterial flagellin protein is the natural ligand of TLR-5 and is known as an effective adjuvant for enhancing immune responses [10, 11]. Virus-like particles (VLPs) are known to be an effective vaccine platform which is egg independent and can elicit both humoral immune response and cellular immune response [11]. In our previous studies, we found that modified flagellin can be expressed effectively in a membrane-bound form and can be incorporated into M1-derived VLPs [12]. We also found that flagellin and four repeats of M2e can be fused together and incorporated into VLPs and induce strong humoral and cellular immune responses [10]. It is known that the central variable region of flagellin is essential for immunogenicity but not necessary for TLR-5 recognition, and the deletion of this region decreases the immunogenicity but retains its mucosal adjuvant function [13–16]. In this study, we designed a membrane-anchored tetrameric M2e protein stabilized by a foreign tetramerization sequence and incorporated the tM2e into influenza virus M1-based VLPs. Chimeric tM2e VLPs containing a truncated flagellin were also produced. We determined whether these VLPs induced broadly protective immunity in mice. 

## 2. Materials and Methods

### 2.1. Ethics Statement

The mice were housed and treated according to Emory University (Atlanta, GA) guidelines and all animal studies were approved by the Emory University Institutional Animal Care and Use Committee (IACUC).

### 2.2. Virus, Peptides, and Cells

*Spodoptera frugiperda* Sf9 cells (Sf9, ATCC, CRL-1711) were maintained in suspension in serum-free SF900 II medium (GIBCO-BRL, Grand Island, NY) at 27°C in spinner flasks at a speed of 80 to 100 rpm. Madin-Darby canine kidney (MDCK) cells were grown and maintained in Dulbecco's modified Eagle's medium (DMEM) plus 10% fetal bovine serum (HyClone, ThermoFisher, Rockford, IL). Mouse-adapted influenza A/Philippine/2/82 (H3N2) and A/PR/8/34 (H1N1) were prepared as lung homogenates from intranasally infected mice and were used for challenge studies. The M2e peptides were synthesized at GenScript (Piscataway, NJ, USA) as shown in Table 1. The purity of the peptide was above 95%. The peptide was dissolved in sterile water and stored at −20°C.

### 2.3. Construction of Membrane-Anchored tM2e and tFliC Genes

A GCN4 sequence-stabilized tetrameric M2e (tM2e) construct was generated as described [17] but with a signal peptide (SP) encoding sequence from honeybee melittin and the transmembrane (TM) and cytoplasmic tail (CT) encoding sequences from the influenza A virus PR8 hemagglutinin (HA) in frame [12]. A membrane-anchored truncated flagellin (tFliC) encoding gene (deletion of variable region) was constructed as described previously [18] but with an influenza HA TM/CT. All constructs were confirmed by DNA sequencing analysis (Eurofins MWG Operon, Huntsville, AL). Recombinant baculoviruses (rBVs) expressing tM2e and tFliC were generated using the Bac-to-Bac protein expression kit (Invitrogen, Grand Island, NY) according to the manufacturer's instruction.

### 2.4. Cell Surface Expression of Membrane-Anchored tM2e

The presence of membrane-anchored tM2e on cell surfaces was determined by a cell surface immune-assay. Two days postinfection with tM2e rBV and mock rBV (rBV expressing human immunodeficiency virus Gag) in 6-well plates, Sf9 cells were washed with phosphate-buffered saline (PBS) at 4°C and then incubated with 1 mL of NHS-SS-biotin dissolved in PBS (0.5 mg/mL) for 30 min at 4°C. Biotinylation was quenched by adding pre-cooled PBS containing 0.1 N glycine. After three washes with precooled PBS, the cells were then lysed with lysis buffer (150 mM NaCl/50 mM Tris HCl, pH 7.5/1 mM ethylenediamine tetraacetate/1% Triton X-100/1% sodium deoxycholate) and precipitated with avidin resins (Fisher scientific Inc., Rockford, IL) at 4°C overnight. After precipitation, resins were washed twice with lysis buffer plus 0.4% SDS and then mixed with 15 *μ*L sample buffer (125 mM Tris-HCl [pH 7.5] containing 4% SDS, 20% glycerol, plus 10% *β*-mercaptoethanol) and heated at 95°C for 5 min before being analyzed by sodium dodecyl sulfate-polyacyrlamide gel electrophoresis (SDS-PAGE).

### 2.5. Preparation of tM2e VLPs

To produce VLPs containing influenza M1 and tM2e (tM2e/M1 VLPs) and truncated flagellin-containing chimeric VLPs (tM2e/tFliC/M1 cVLPs), Sf9 cells were coinfected with rBVs expressing M1 and tM2e, tFliC, tM2e and M1, or tFliC and M1, respectively. The multiplicities of infection (MOIs) of rBVs expressing tM2e, M1 were adjusted to 4 : 2 to produce tM2e VLPs, and the MOIs of rBVs expressing tFliC, tM2e, M1 were adjusted to 1 : 4 : 2 to produce chimeric tFliC-containing tM2e VLPs (cVLPs). The quality of the purified VLPs was determined by Western blotting analysis and immunoelectron microscopic observation. The sterility was determined by inoculating LB medium and incubating the culture at 37°C for 48 h. 

### 2.6. Cell Surface Immunogold Labeling and Electron Microscopy of Infected Cells

At 60 hr postinfection, Sf9 cells were washed with 0.3 M HEPES containing 0.02 M lysine and were blocked in 0.2 M HEPES-1.5% bovine serum albumin (BSA) for 60 min at 4°C. After blocking, cells were incubated with mouse anti-M2e antibody (Abcam Inc., Cambridge, MA) at a 1 : 100 dilution at 4°C for 60 min. After washing, a 10 nm gold-conjugated goat anti-mouse antibody (Sigma-Aldrich, MO, USA) was added at a dilution of 1 : 100 for another 60 min. Cells were then washed and fixed with 1% glutaraldehyde in PBS for 30 min, incubated with 1% osmium tetroxide for 1 hour, and then stained with tannic acid. The samples were dehydrated and embedded in EMBED 812 (Electron Microscopy Sciences, Ft. Washington, PA). Samples were then stained with uranyl acetate and lead citrate and examined with a Philips (Mahwah, NJ) CM 10 electron microscope at the Robert P. Apkarian Integrated Electron Microscopy Core of Emory University.

### 2.7. Immunization and Challenge

For animal experiments, 6–8 weeks old female BALB/c mice (Harlan Laboratories, Indianapolis, IN) were intranasally (i.n.) immunized three times with 0.5 *μ*g soluble tM2e protein, tM2e VLPs (tM2e VLP), tM2e/tFliC VLPs, a mixture of tM2e VLPs and tFliC VLPs, a mixture of tM2e VLPs and soluble flagellin protein with 0.5 *μ*g M2e content, or 0.5 *μ*g soluble flagellin protein only at 4-week intervals. Each group included 18 mice. Four weeks after the final immunization, mice were challenged with a lethal dose of A/PR/8/34 (5 × 50% mouse lethal dose (5 LD50)) or A/Philippines/82 influenza virus (5 LD50). Mice were monitored daily to record body weight changes and mortality using 25% loss in body weight as the Institutional Animal Care and Use Committee (IACUC) endpoint.

### 2.8. Sample Collections

Blood samples were collected at one week before immunization for preimmune sera and 2 weeks after each immunization for immune sera by retro-orbital plexus puncture. Sera were collected by a brief spin (5000 rpm for 5 min) after clotting (about 2 hours at room temperature). Mucosal samples were collected before challenge and 4 days after challenge. Nasal washes were collected by lavaging mouse nostrils repetitively with 1 mL of PBS containing 0.05% Tween 20 (PBST). For lung washes, individual mouse lungs were lavaged repetitively with 1 mL PBST. After a brief centrifugation (8000 rpm) for 10 min, supernatants were filtered through a 0.22 *μ*m filter and stored at −80°C for further assays. Lymphocytes from lung and spleen samples were collected from mice sacrificed 4 weeks after the final boost and were used for ELISPOT as described previously [12].

### 2.9. Antibody ELISA

M2e specific antibody (Ab) titers in immune sera were determined by ELISA as described previously [10, 11] using synthesized M2e peptides (1 *μ*g/mL) as coating antigens. The highest dilution which gives an OD_450_ of at least twice the standard deviation of that of the naïve group at the same dilution was designated as the Ab endpoint titer. To evaluate the cross-reactivity to variant M2e-sequence peptides, immune sera were tested for binding to M2e peptides derived from Phi/82, PR/8, CA/09, and VIET/04, respectively, by ELISA. The optical density at 450 nm was used to compare the level of reactivity.

Antibodies recognizing native M2 protein were determined by using cell surface ELISA. M2-expressing MDCK cells were maintained in DMEM media with 7.5 *μ*g/mL of puromycin (Invitrogen, Carlsbad, CA), 5 *μ*M of amantadine (Sigma, St. Louis, MO) and 10% FBS (Invitrogen, Carlsbad, CA) at 37°C in 5% CO_2_. Confluent M2-expressing MDCK monolayer cells were fixed by 0.05% glutaraldehyde and 10% buffered formalin (Sigma, St. Louis, MO) for 30 min at room temperature and were used to determine Ab levels binding to M2 expressed on cell surfaces by ELISA as described [12]. 

### 2.10. Cytokine ELISpot

Interferon gamma (INF-*γ*) and interleukin 4 (IL-4) secreting T cells from immunized mouse splenocytes or lung cells were evaluated using ELISpot kits (eBioscience, San Diego, CA) according to the manufacturer's instructions [12]. Anti-mouse IFN-*γ* or IL-4 Abs was used to coat Multiscreen 96-well filtration plates (Millipore, Billerica, MA). A volume of 100 *μ*L freshly isolated splenocytes (1 × 10^7^ cells/mL) were added to each well and stimulated with M2e peptide (10 *μ*g/mL). The plates were incubated for 40 h at 37°C with 5% CO_2_. The development and counting of ELISpot were performed following the manufacturer's procedures.

### 2.11. Lung Viral Titers

 Whole lungs were collected at day 4 postinfection (p.i.) and ground using cell strainers (BD Falcon, Franklin Lakes, NJ). Lung homogenates were centrifuged at 1000 RPM for 10 min to remove tissue debris. MDCK cell-based plaque assay was used for lung virus titration as described previously.

### 2.12. Statistical Analysis

Comparisons among vaccinated groups were performed using a two-tailed Student's *t*-test. The analyses were done by using GraphPad Prism version 5.00 for Windows (GraphPad Software, San Diego, CA). P values of less than 0.05 (*P* < 0.05) were considered to be statistically significant. 

## 3. Results

### 3.1. Preparation and Characterization of VLPs

**Figure 1:**
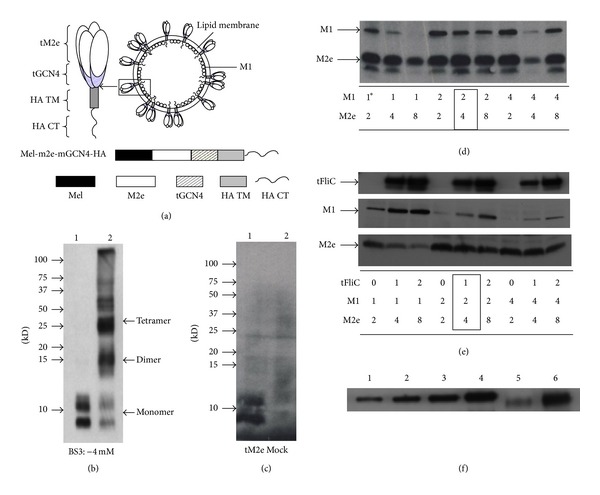
Generation and characterization of tM2e VLPs. (a) Diagram of tM2e construct. (b) Tetramerization of tM2e in VLPs. M2e VLPs were produced as described in Material, and Methods. One *μ*g of tM2e VLPs was cross-linked with BS3 at concentrations of 4 mM. Cross-linked tM2e VLPs samples were applied to Western blot and probed using mouse monoclonal antibody 14C2 (Abcam Inc., Cambridge, MA). Lane 1: without BS3. Lane 2: with 4 mM BS3. (c) Cell surface expression of membrane-anchored tM2e. Surface expression of the membrane-anchored tM2e was detected by cell surface biotinylation. Lane 1: Cell lysate from cells infected with rBV expressing membrane-anchored tM2e; Lane 2: mock rBV (rBV expressing human immunodeficiency virus Gag)-infected cells. ((d), (e)) Optimization of VLP production: Sf9 cells were infected with rBVs expressing tM2e, M1, and tFliC at different MOIs as designated at the bottom. VLPs were prepared as described in Materials and Methods. The resulting VLPs were analyzed by Western blotting. tM2e and M1 bands were probed with mouse monoclonal antibody 14C2 and mouse monoclonal anti-M1 antibody. Membrane-anchored truncated flagellin (tFliC) was probed with guinea pig anti-flagellin polyclonal antibody. The asterisk means MOIs of different virus. The best ratio was framed in the figure. (f) Western blotting of tM2e VLP and recombinant M2e protein. Recombinant M2e protein (Lanes 1, 2, and 3 and 4, 15, 30, 60, and 120 ng, resp.), tM2e VLPs (Lane 6: 5 *μ*g total protein) and tM2e/tFliC VLPs (Lane 5: 5 *μ*g totoal protein) were loaded and detected by Western blot using mouse anti-M2e monoclonal antibody (14C2). Amount of M2e protein incorporated in tM2 VLPs was calculated by spot densitometry analysis using serial diluted recombinant tM2e protein as a standard.

**Figure 2:**
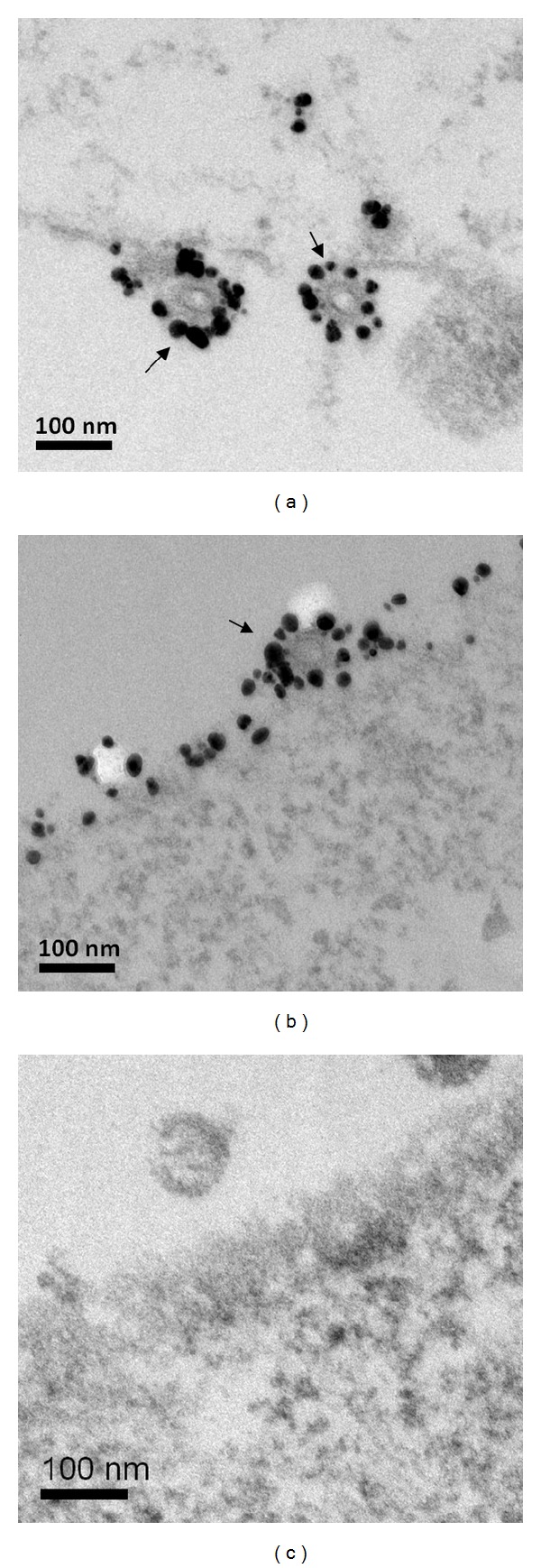
Cell surface immunogold labeling and electron microscopy of infected cells. Electron microscopy of thin sections of cells co-infected with (a) rBVs expressing tM2e and M1; (b) rBVs expressing tFliC, tM2e, and M1; or (c) rBVs expressing M1 only. Surface immunolabeling was done prior to fixation, embedding, and sectioning of the infected cells at 48 h postinfection. The primary antibody was a mouse anti-M2e antibody at 1 : 100 dilution, and the secondary antibody was 10 nm gold-conjugated goat anti-mouse antibody at 1 : 100 dilution. The particles show a prominent layer of surface spikes (bar = 100 nm).

To improve the immunogenicity of M2e, a modified tetrameric M2e (tM2e) construct was designed as showed in Figure 1(a). A modified flagellin with a central variable region truncation and a membrane anchor from influenza HA was designed as a molecular adjuvant, as described before with slight modifications [18]. To determine the role of tM2e in inducing cross protection against heterologous virus, we produced influenza VLPs containing the tM2e protein. To confirm the tetrameric structure of tM2e after expression and purification, we used the chemical cross-linker Bis[sulfosuccinimidyl] suberate (BS3) to fix its polymeric state. Following cross-linking, SDS-PAGE analysis revealed a major band with a molecular mass of 28 kDa representing the M2e tetramer and a band with a molecular mass of 14 kDa representing the dimers (Figure 1(b), lane 2). We observed only one band with a molecular mass of 7 kDa representing the M2e monomer without the addition of BS3 (Figure 1(b), lane 1). As shown in Figure 1(c), lane 1, tM2e was expressed well on the surface of insect cells, demonstrating that the GCN4-stabilized tM2e was expressed in a tetrameric form and could be transported onto the cell surface [17]. The lower band in Figure 1(b) is a degradation product, same as the band on Figure 1(d) under M2e. The incorporation of tM2e or tFliC into M1 VLPs was confirmed by Western blot using anti-M2 monoclonal antibody 14C2, anti-flagellin polyclonal antibodies, or anti-M1 polyclonal antibodies (Figures 1(d) and 1(e)). The M2e content in VLPs was evaluated using Western blotting as described [19]. Recombinant M2e protein (Lanes 1, 2, and 3 and 4, 15, 30, 60, and 120 ng, resp.) was used as standard, and tM2e VLPs (Lane 6: 5 *μ*g total protein) and tM2e/tFliC VLPs (Lane 5: 5 *μ*g total protein) were loaded and detected. Amount of M2e protein incorporated in M2e VLP was calculated by spot densitometry analysis using serial diluted rM2e protein as a standard as shown in Figure 1(f) using Western blotting as described [19]. We also use soluble flagellin as standard and check the tFlic concentration in tFlic VLPs and used same dose as soluble flagellin. Furthermore, the presence of tM2e in the VLPs and tM2e VLP budding from sf9 cell membrane were confirmed by examining sections of cell surface after immunogold labeling as shown in Figure 2. Both tM2e VLPs and tM2e/tFliC VLPs showed spherical shapes with gold patches on their surfaces, demonstrating the incorporation of tM2e into VLPs and tM2e VLP budding from insect cells. 

### 3.2. Tetrameric M2e VLPs Induced Strong Humoral Responses

**Figure 3:**
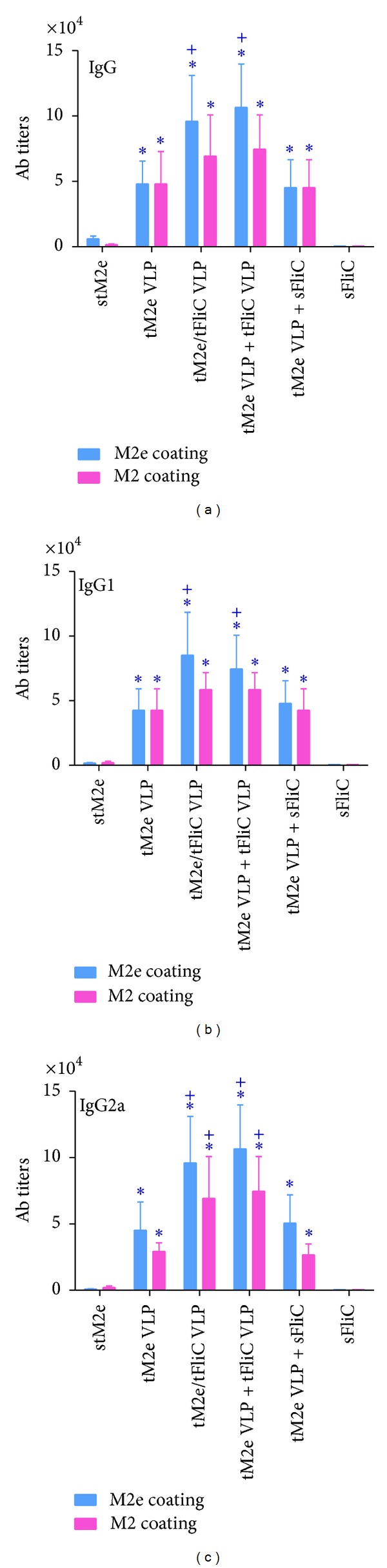
Serum IgG and isotype endpoint titers. Serum antibodies specific for M2e peptide and MDCK expressed M2 were determined. The highest dilution that gave an OD450 of twice that of the naive group at the lowest dilution was designated as the Ab endpoint titer. G1: Soluble tetrameric M2e protein (stM2e), G2: tM2e VLPs; G3: tM2e/tFliC VLPs; G4: tM2e VLP + tFliC VLPs; G5: tM2e VLP + soluble flagellin; G6: soluble flagellin. (a) Serum IgG; (b) IgG1; (c) IgG2a. Each group has 6 mice. The asterisk (∗) indicates a significant difference between G2 and G1, G3 and G1, G4 and G1, and G5 and G1. The “+” indicates a significant difference between G3 and G2, G4 and G2, and G5 and G2.

To determine if i.n. immunization with tM2e VLPs could induce enhanced humoral responses, immune sera were evaluated for antigen-specific IgG titers using ELISA with M2e peptides as coating antigens, or cell surface ELISA using M2-expressing MDCK cells. As shown in Figure 3(a), M2e-specific IgG endpoint titers in sera of G2 (tM2e VLPs), G3 (tM2e/tFliC VLPs), G4 (tM2e VLPs + tFliC VLPs), and G5 (tM2e VLPs + soluble flagellin) were approximately 10- to 30-fold higher than that of the mouse group immunized with only soluble tM2e (*P* < 0.05) (Figure 3(a)). The levels of M2e-specific IgG1 and IgG2a were also significantly higher than those of mice receiving only tM2e (Figures 3(b) and 3(c)). Flagellin showed a significant adjuvant effect on the magnitude of M2e-specific Ab responses when either integrated into tM2e VLPs or when tFliC VLPs were mixed with tM2e VLPs. As shown in Figure 3, the levels of IgG, IgG1, or IgG2a in sera of G3 (tM2e/tFliC VLP) and G4 (mixture of tM2e VLPs and tFliC VLPs) were significantly higher than G2 which was immunized with tM2e VLPs alone. To provide more details of the comparison of the antibody titer between each group, endpoint titers of each group were shown in Supplemental Material (Tables 1–4), and the *P* values between different groups were also shown in Supplemental Material (Tables 5–8) (See Supplementary Material available online at http://dx.doi.org/10.1155/2013/686549). The flagellin-containing VLPs (G3 and G4) elicited higher levels of IgG2a (IgG1/IgG2a ratio, 0.89 and 0.7, resp.) compared to tM2e VLPs (G2) (IgG1/IgG2a ratio, 1.23). Immune sera also showed similar binding reactivity to native M2 expressed on MDCK cell surfaces, suggesting that antibodies induced by tM2e VLPs can recognize conformational epitopes on native M2. These data show that tM2e VLPs can elicit strong humoral responses and that membrane-anchored truncated flagellin shows a strong adjuvant effect.

**Figure 4:**
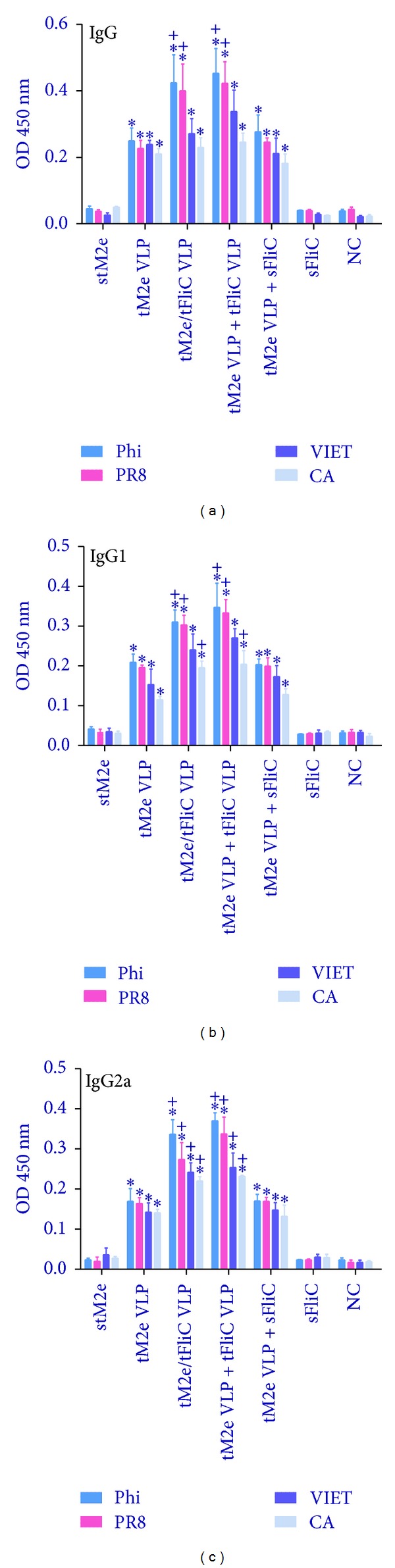
Cross-reactivity to variant M2e peptides. The cross-reactivity of immune sera to variant M2e peptides was assessed by ELISA. G1: Soluble tetrameric M2e protein (stM2e), G2: tM2e VLPs; G3: tM2e/tFliC VLPs; G4: tM2e VLP + tFliC VLPs; G5: tM2e VLP + soluble flagellin; G6: soluble flagellin; NC: naive group. (a) Serum IgG; (b) IgG1; (c) IgG2a. Each group has 6 mice. The asterisk (∗) indicates a significant difference between G2 and G1, G3 and G1, G4 and G1, and G5 and G1. The “+” indicates a significant difference between G3 and G2, G4 and G2, and G5 and G2.

 Although M2e is highly conserved in influenza A viruses, some amino acid substitutions occur in different strains and subtypes. To evaluate the cross-reactivity of M2e-specific Abs induced by tM2e VLPs, sera from mice immunized i.n. with tM2e VLPs were assayed using ELISA with variant M2e peptides as coating antigens. As shown in Table 1, the M2e sequence in tM2e VLPs is the same in human viral M2e consensus and in Phi/82 H3N2 M2e, whereas there are 1, 4, and 3 amino acid differences, respectively, in M2e of PR/8, CA/09 and VIET/04 viruses. As demonstrated in Figure 4, M2e Abs bound strongly to the M2e peptides of Phi/82 and PR/8. The levels of Ab binding were significantly lower in VIET/04 and CA/09 M2e peptides (*P* < 0.05). These results demonstrate that the assembly of the tM2e antigen into VLPs can significantly enhance the immunogenicity of tM2e and can induce M2e-specific antibodies with some cross-reactivity to variant M2e sequences. These results also show that when M2e and flagellin are combined together in VLPs (G3) or tM2e VLPs and flagellin VLPs are mixed together (G4), systemic responses were enhanced more than tM2e VLPs mixed with soluble flagellin protein after immunization (G5), which indicate that the membrane-anchored truncated flagellin shows a stronger adjuvant effect than the soluble form in induction of serum IgG responses.

### 3.3. Tetrameric M2e VLPs Induce Robust Mucosal Antibody Responses

**Figure 5:**
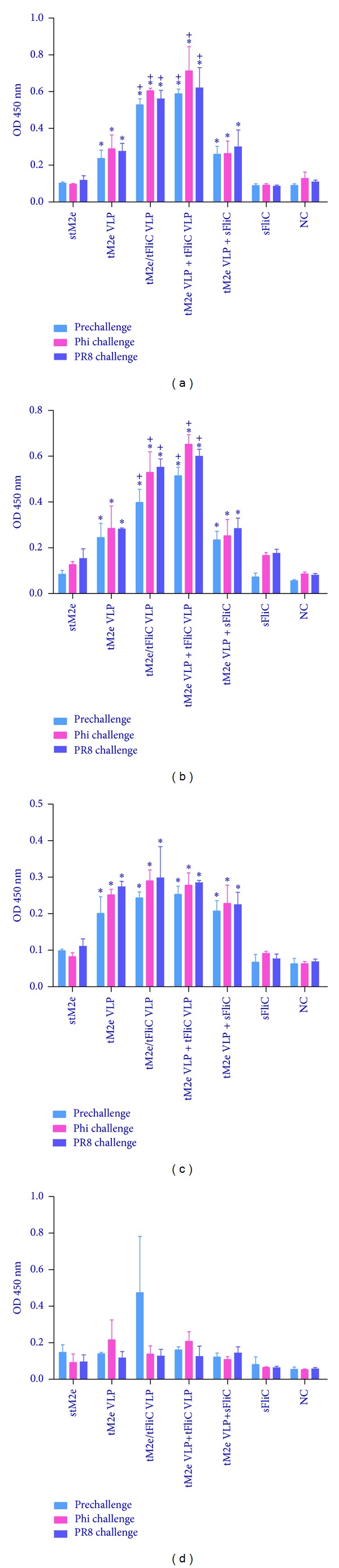
Mucosal binding antibody levels. Lung and nasal washes were collected from individual mice before and at day 4 postchallenge with A/Philippines/82 (H3N2) and A/Puerto Rico/8/34 (H1N1) viruses. Lung and nasal washes, with 5-fold dilution and no dilution, respectively, were used to determine IgA and IgG antibody binding to M2e peptide in ELISA. (a) IgA binding in lung washes. (b) IgG binding in lung washes. (c) IgA binding in nasal washes. (d) IgG binding in nasal washes. Each group has 3 mice. The asterisk (∗) indicates a significant difference between G2 and G1, G3 and G1, G4 and G1, and G5 and G1. The “+” indicates a significant difference between G3 and G2, G4 and G2, and G5 and G2.

A strong mucosal antibody response is associated with the prevention of viral entry in the upper respiratory tract of mice [20]. To evaluate whether i.n. immunization with tM2e VLPs could induce mucosal Ab responses, we measured M2e-specific IgA and IgG Abs from lung and nasal washes by ELISA in mice prechallenge and 4 days after challenge (Figure 5). As shown in Figures 5(a) and 5(b), significantly higher levels of IgG and IgA were observed in lung washes of prechallenged mice of G2 (tM2e VLP), G3 (tM2e/tFliC VLP), G4 (tM2e VLP + tFliC VLP), and G5 (tM2e VLP + soluble flagellin) compared to G1 which received only soluble tM2e (*P* < 0.05). Furthermore, groups G3 and G4 have significantly higher levels of IgG and IgA compared to G2 which received only tM2e VLPs. After challenges with Phi/82 or PR8 virus, the lung wash IgG and IgA levels increased, as well as nasal wash IgA, and there was no significant IgG change between prechallenge and postchallenge nasal washes (Figure 5). Similarly, significantly higher levels of IgA were observed in nasal washes of prechallenge or postchallenge mice immunized with tM2e VLP, tM2e/tFliC VLP, tM2e VLP + tFliC VLP, or tM2e VLP + soluble flagellin (*P* < 0.05). These data demonstrate that tM2e VLPs stimulate M2e-specific mucosal Ab responses in mice, and incorporating the membrane-anchored flagellin in VLPs as adjutants can further stimulate mucosal antibody secretion.

### 3.4. Tetrameric M2e VLPs Activate M2e-Specific T-Cell Responses

**Figure 6:**
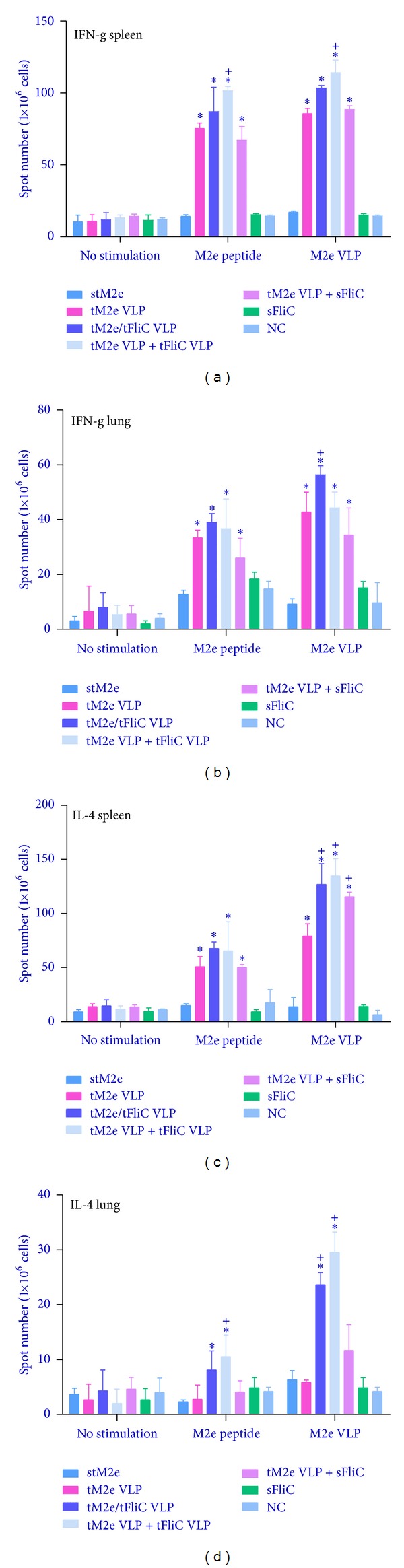
Cellular immune responses. Cellular immune responses were assessed with cells from spleens and lungs of immunized mice. Cells from spleens ((a), (c)) and lungs ((b), (d)) were stimulated with M2e peptides and M2e VLPs for 40 hours, and cytokine-secreting cell colonies were determined by ELISPOT assay. Each group has 3 mice. The asterisk (∗) indicates a significant difference between G2 and G1, G3 and G1, G4 and G1, and G5 and G1. The “+” indicates a significant difference between G3 and G2, G4 and G2, and G5 and G2.

T-cell responses are important for the generation and regulation of an effective immune response and are known to contribute to broad cross-protective immunity [21, 22]. IFN-*γ* and IL-4 secreting cells in both the spleens and lungs of immunized mice were evaluated by cytokine ELISpot as described previously [11]. As shown in Figure 6, mice of groups G2, G3, G4, or G5 showed significantly higher IFN-*γ* secreting cell populations in the spleens and lungs after stimulation with M2e peptide or tM2e VLPs compared with mice immunized with soluble tM2e (Figures 6(a) and 6(b), *P* < 0.05). A higher frequency of IL-4 secreting cells was also detected in the spleens of immunized mice in G2, G3, G4, or G5 compared to that of mice vaccinated with soluble tM2e (Figure 6(c), *P* < 0.05). However, only G2, G3, and G4 had higher frequencies of IL-4 secreting cells in the lungs after stimulation with tM2e VLPs and only background levels of cytokine secreting cells were detected after M2e peptide stimulation (Figure 6(d)). These results provide evidence that i.n. immunization with tM2e VLPs induces enhanced M2e specific T-cell immune responses. 

### 3.5. Tetrameric M2e VLPs Protect Mice from Lethal Virus Challenge

To evaluate the protective efficacy of tM2e VLPs, immunized mice were infected with 5LD_50_ of Phi/82 H3N2 virus or PR/8 H1N1 virus 4 weeks after the final boost immunization. Mouse body weight loss and survival were monitored for 14 days. Day 4 postinfection was chosen for evaluation of lung virus titers because naive mice were previously shown to have substantial titers of virus in the lungs at that time point [23]. 

**Figure 7:**
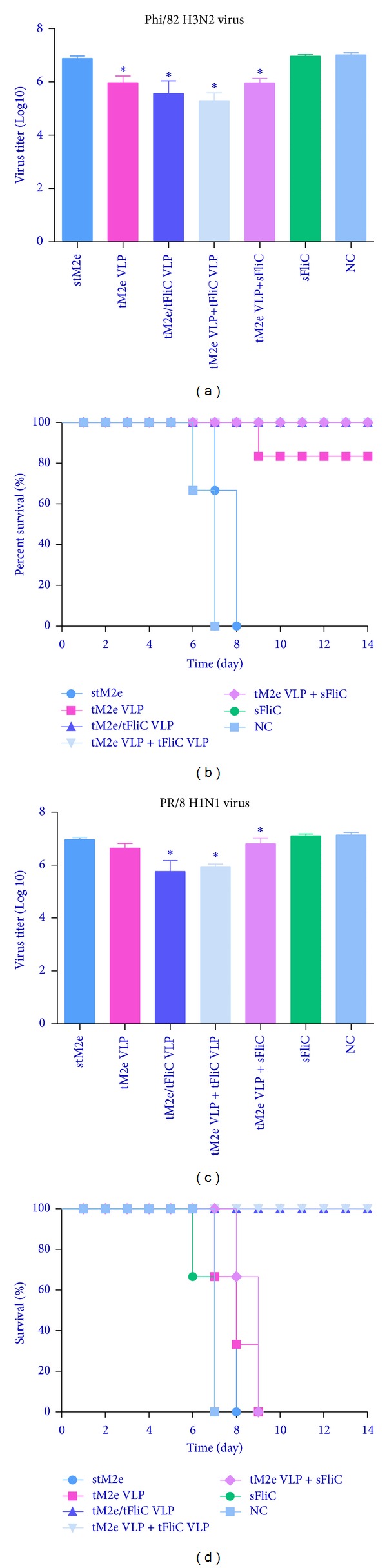
Live virus challenges with A/Philippines/82 and A/Puerto Rico/8/34 strains. Immunized mice were challenged with a lethal dose (5LD50) of A/Philippines/82 or A/Puerto Rico/8/34 in a volume of 30 *μ*L PBS by i.n. instillation. Each group has 3 mice. Mouse survival rate and body weight changes were monitored daily for 14 days. Each group has 3 mice. Mouse lung virus titers at day 4 postchallenge were determined by a standard plaque assay. Each lung sample was grinded, cleared, and diluted in 2ml DMEM. Bars represent mean virus titers (Log10 pfu/mL) ± standard errors from three independent assays. The asterisk (∗) indicates a significant difference between group immunized with stM2e only and other groups, (*P < 0.05). (a) and (b) Lung virus titers at day 4 and survival rate after A/Phi/82 virus infection; (c) and (d) lung virus titers at day 4 and survival rate after PR/8 infection.

As shown in Figure 7, all mice immunized with tM2e protein alone, soluble flagellin protein alone or naive groups died by 7 to 9 days p.i. (Figures 7(b), and 7(d)) with high titers of viruses in the lungs (Figures 7(a) and 7(c)). G1 (tM2e protein), G2 (tM2e VLP), G5 (tM2e VLP + soluble flagellin), and G6 (soluble flagellin protein) did not show substantial virus reduction in the lungs compared with the control (*P* > 0.05). In contrast, mice immunized with tM2e/tFliC VLPs or tM2e VLP + tFliC VLPs had significant lower virus titers in their lungs after Phi/82 or PR/8 challenge (*P* < 0.05) and exhibited reduced morbidity compared with mice in the tM2e protein group. All mice in G3 and G4 survived the lethal challenge with homologous Phi/82 H3N2 virus or heterosubtypic PR/8 H1N1 virus. These results indicate that i.n. immunization of mice with tM2e VLPs with flagellin as an adjuvant significantly reduces virus titers in the lungs and completely protects mice against severe disease and death from lethal infection with the homologous virus or heterosubtypic virus.

## 4. Discussion

M2e is considered to be a promising target for inducing cross-protection because it is highly conserved among influenza A viruses [5]. However, the immunogenicity of M2e is very low due in part to its low incorporation level into influenza virus particles and its small ectodomain, which may be sterically blocked by HA and NA on the surface of the virus. These properties of native M2e limit its ability to be sensed by host immune cells. In this study, we found that tM2e was incorporated into virus-like particles at high levels without the presence of HA and NA and thus could be delivered to and effectively sensed by immune cells. Since antibodies specific to conformational epitopes presented in quaternary structures may be more effective at binding native M2 [8], a tetrameric conformation-stabilized recombinant M2e was predicted to be more immunogenic than other M2e forms. The tetrameric M2e that we produced was stabilized by the addition of tGCN4 [17] and a C-terminal membrane anchor from the A/PR8 influenza virus HA [10] and was expressed efficiently in a recombinant baculovirus protein expression system in insect cells. Our results demonstrated that intranasal vaccination with tM2e VLPs induces high levels of humoral and mucosal M2e-specific antibody and T-cell responses compared to those observed with the soluble tM2e. Furthermore, these Abs were shown to recognize native M2 on cell surfaces.

VLPs is an effectve platform to increase the immunogenicity of antigen [10]. Besides, we have observed that the TLR5 ligand flagellin can be incorporated into influenza VLPs and is an effective adjuvant for preventing mucosal infection of influenza viruses [11, 12]. However, there are questions about whether the immunogenicity of flagellin is a disadvantage for its use as an adjuvant [12, 24]. Considering these, we modified flagellin by truncating the variable region which is essential for immunogenicity but not necessary for its TLR5 binding activity and incorporated it into influenza tM2e VLPs by fusing a membrane-anchoring sequence from HA. We found that the membrane-anchored truncated flagellin shows a stronger adjuvant effect than the soluble form in induction of humoral immune responses when animals were immunized intranasally. The same amount of soluble flagellin also showed reduced adjuvant effect when compared with tFliC VLPs, which suggest that different mechanisms may be involved in these two different forms. 

Flagellin can promote murine antibody responses by either membrane receptor TLR5-mediated activation of NF-*κ*B or cytosolic receptor NLRC4-mediated activation of the inflammasome [25]. VLPs are similar to native virus in size and can enter cells to release contents of the core-like particle into the cytoplasm of the cell [11]. Flagellin-containing M2e VLPs can enter cells, react with the flagellin cytosolic receptor NLRC4 (also referred to as IPAF), and drive rapid generation of inflammasome cytokines such as IL-1*β* and IL-18 to provide a sufficient second signal. The cytosolic innate signaling drives the T-cell proliferation necessary to generate antibodies to both flagellin itself and a coadministered antigen [25]. Soluble flagellin can recognize flagellin receptor TLR5 on the cell surface and activate NF-*κ*b [25]. In our experimental groups, soluble flagellin administrated with M2e VLP could protect mice from homologous virus infection but not heterosubtypic virus infection, whereas flagellin-containing M2e VLPs could protect mice from both homologous and heterosubtypic virus infections. This phenomenon suggests that NLRC4 dependent pathway may play a dominant role in the promotion of antibody responses by flagellin-containing VLPs. Furthermore, we found that tFliC-containing VLPs preferentially activated Th1-responses with a lower IgG1/IgG2a ratio, which is similar to the membrane-anchored full-length flagellin and flagellin in its native surface-bound context on live Salmonella [12, 26]. However, the mechanism of tFliC on Ig isotype switching is still unknown. 

Our aim was to combine the advantage of VLPs as a vaccine platform and flagellin as an adjuvant to increase the immunogenicity of the conserved M2e and induce cross-protective immunity to homo-/heterologous influenza viral infections. From our challenge data we can see that even with high level of Ab titers, M2e VLPs induce limited protection from heterosubtypic virus infection. One possible reason is that so far only a single human CTL epitope has been identified for M2e inducing memory CTL activity [27], and M2e specific antibody does not have neutralization effect but they have weak protection via antibody-dependent NK cell activity [28]. In fact, in our experiment, tFlic-containing VLPs could enhance immune response via the NLRC4 pathway since VLPs can enter into the cells. So the antibody-dependent cell-mediated cellular cytotoxicity might be involved in the protective effect of heterosubtypic virus infection.

The mucosal immune system is the first immunological barrier against viruses that invade the body via the mucosal surface [29]. M2e-based vaccines were previously reported to induce mucosal immune response when delivered by the i.n. route [30–32]. Several groups demonstrated that IgA in upper respiratory tract secretions plays a major role in antiviral immunity [33–35]. The local production of IgG is also an important component of the immune responses following mucosal immunization or infection [36, 37]. We found that high levels of sIgA and IgG were induced in both nasal mucosal and lung surfaces after i.n. administration of tM2e VLPs. The consistency of these antibody responses to the observed protection indicates that mucosal antibodies may be one of the immune correlates of protection. A combination of enhanced mucosal immunity and antibody-dependent cell-mediated cellular cytotoxicity may be protecting mice from lethal challenge from homo/hetero strains.

Although M2e is relatively conserved in all influenza A viruses, variation of M2e sequences in different viruses may indicate the different protection efficacy of M2e vaccines to various virus infection. In our study, tM2e VLPs showed complete protection of mice from Phi/82 (H3N2) virus infection; it still cannot protect mice from PR/8 (H1N1) virus infection which may be associated with the 1-aa substitutions in the M2e of PR/8. Indeed, our ELISA binding assay illustrated that even a 1-aa variation could impact the binding avidity of M2e-specific Abs with synthesized peptides. However, it is noteworthy that tFlic-containing VLPs showed complete protection of mice from either Phi/82 (H3N2) virus infection or PR/8 (H1N1) virus infection. Therefore, it is worth to further investigate those strategies that could enhance the cross-protection of M2e vaccines, for instance, incorporating representative M2e sequences from different subtypes into one construct, or coexpressing with HA or NA antigens, as well as combining with better adjuvant strategy. 

## 5. Conclusions

M2e is one of the target antigens for a universal vaccine against influenza. Strategies like administration in naturally conformational form carried by VLPs and adjuvanted by bioactive factors demonstrate significant improvement of both immunogenicity and protective efficacy of M2e. Our results support the promising application of tM2e cVLP-based universal vaccines and warrant further investigation.

## Supplementary Material

The supplemental material provided includes:
(1) Table 1 includes the endpoint IgG titer of each group using ELISA with M2e peptides as coating antigens.
(2) Table 2 includes the endpoint IgG1 titer of each group using ELISA with M2e peptides as coating antigens.
(3) Table 3 includes the endpoint IgG2a titer of each group using ELISA with M2e peptides as coating antigens.
(4) Table 4 includes the endpoint IgG2a titer of each group using cell surface ELISA with M2-expressing MDCK cells.
(5) Table 5 includes the P values of IgG titer between different groups when using ELISA with M2e peptides as coating antigens.
(6) Table 6 includes the P values of IgG1 titer between different groups when using ELISA with M2e peptides as coating antigens.
(7) Table 7 includes the P values of IgG2a titer between different groups when using ELISA with M2e peptides as coating antigens.
(8) Table 8 includes the P values of IgG2a titer between different groups when using cell surface ELISA with M2-expressing MDCK cells.

## Figures and Tables

**Table 1:** M2e amino acid of influenza A virus.

Viral strains	Subtype	M2e amino acid sequence
M2e in VLPs	N/A*	MSLLTEVETP IRNEWGCRCN D
A/Philippines/2/82	H3N2	MSLLTEVETP IRNEWGCRCN D
A/Puerto Rico/8/34	H1N1	MSLLTEVETP IRNEWGCRCN **G**
A/California/04/09	H1N1	MSLLTEVETP **T**R**S**EW**E**CRC**S** D
A/Vietnam/1203/04	H5N1	MSLLTEVETP **T**RNEW**E**CRC**S** D

*M2e consensus of human influenza A viruses.

## References

[1] de Wit E, Fouchier RAM (2008). Emerging influenza. *Journal of Clinical Virology*.

[2] Smith GJD, Vijaykrishna D, Bahl J (2009). Origins and evolutionary genomics of the 2009 swine-origin H1N1 influenza a epidemic. *Nature*.

[3] Tite JP, Hughes-Jenkins C, O’Callaghan D (1990). Anti-viral immunity induced by recombinant nucleoprotein of influenza A virus. II. Protection from influenza infection and mechanism of protection. *Immunology*.

[4] Epstein SL, Kong W-P, Misplon JA (2005). Protection against multiple influenza A subtypes by vaccination with highly conserved nucleoprotein. *Vaccine*.

[5] Schotsaert M, De Filette M, Fiers W, Saelens X (2009). Universal M2 ectodomain-based influenza A vaccines: preclinical and clinical developments. *Expert Review of Vaccines*.

[6] De Filette M, Fiers W, Martens W (2006). Improved design and intranasal delivery of an M2e-based human influenza A vaccine. *Vaccine*.

[7] Wolf AI, Mozdzanowska K, Williams KL (2011). Vaccination with M2e-based multiple antigenic peptides: characterization of the B cell response and protection efficacy in inbred and outbred mice. *PLoS ONE*.

[8] Feng J, Zhang M, Mozdzanowska K (2006). Influenza A virus infection engenders a poor antibody response against the ectodomain of matrix protein 2. *Virology Journal*.

[9] Steinhagen F, Kinjo T, Bode C, Klinman DM (2011). TLR-based immune adjuvants. *Vaccine*.

[10] Wang BZ, Gill HS, Kang SM (2012). Enhanced influenza virus-like particle vaccines containing the extracellular domain of matrix protein 2 and a Toll-like receptor ligand. *Clinical and Vaccine Immunology*.

[11] Wang B-Z, Xu R, Quan F-S, Kang S-M, Wang L, Compans RW (2010). Intranasal immunization with influenza VLPs incorporating membrane-anchored flagellin induces strong heterosubtypic protection. *PLoS ONE*.

[12] Wang B-Z, Quan F-S, Kang S-M, Bozja J, Skountzou I, Compans RW (2008). Incorporation of membrane-anchored flagellin into influenza virus-like particles enhances the breadth of immune responses. *Journal of Virology*.

[13] Yoshioka K, Aizawa S-I, Yamaguchi S (1995). Flagellar filament structure and cell motility of Salmonella typhimurium mutants lacking part of the outer domain of flagellin. *Journal of Bacteriology*.

[14] Andersen-Nissen E, Smith KD, Strobe KL (2005). Evasion of Toll-like receptor 5 by flagellated bacteria. *Proceedings of the National Academy of Sciences of the United States of America*.

[15] Smith MF, Mitchell A, Li G (2003). Toll-like receptor (TLR) 2 and TLR5, but not TLR4, are required for *Helicobacter pylori*-induced NF-*κ*B activation and chemokine expression by epithelial cells. *Journal of Biological Chemistry*.

[16] Nempont C, Cayet D, Rumbo M, Bompard C, Villeret V, Sirard J-C (2008). Deletion of Flagellin’s hypervariable region abrogates antibody-mediated neutralization and systemic activation of TLR5-dependent immunity. *Journal of Immunology*.

[17] De Filette M, Martens W, Roose K (2008). An influenza A vaccine based on tetrameric ectodomain of matrix protein 2. *Journal of Biological Chemistry*.

[18] Vassilieva EV, Wang B-Z, Vzorov AN (2011). Enhanced mucosal immune responses to HIV virus-like particles containing a membrane-anchored adjuvant. *MBio*.

[19] Song J-M, Wang B-Z, Park K-M (2011). Influenza virus-like particles containing M2 induce broadly cross protective immunity. *PLoS ONE*.

[20] Ito R, Ozaki YA, Yoshikawa T (2003). Roles of anti-hemagglutinin IgA and IgG antibodies in different sites of the respiratory tract of vaccinated mice in preventing lethal influenza pneumonia. *Vaccine*.

[21] Kreijtz JHCM, Bodewes R, van Amerongen G (2007). Primary influenza A virus infection induces cross-protective immunity against a lethal infection with a heterosubtypic virus strain in mice. *Vaccine*.

[22] Sang Heui Seo SHS, Webster RG (2001). Cross-reactive, cell-mediated immunity and protection of chickens from lethal H5N1 influenza virus infection in Hong Kong poultry markets. *Journal of Virology*.

[23] Lu X, Tumpey TM, Morken T, Zaki SR, Cox NJ, Katz JM (1999). A mouse model for the evaluation of pathogenesis and immunity to influenza A (H5N1) viruses isolated from humans. *Journal of Virology*.

[24] Honko AN, Sriranganathan N, Lees CJ, Mizel SB (2006). Flagellin is an effective adjuvant for immunization against lethal respiratory challenge with Yersinia pestis. *Infection and Immunity*.

[25] Vijay-Kumar M, Carvalho FA, Aitken JD, Fifadara NH, Gewirtz AT (2010). TLR5 or NLRC4 is necessary and sufficient for promotion of humoral immunity by flagellin. *European Journal of Immunology*.

[26] Cunningham AF, Khan M, Ball J (2004). Responses to the soluble flagellar protein FliC are Th2, while those to FliC on Salmonella are Th1. *European Journal of Immunology*.

[27] Gianfrani C, Oseroff C, Sidney J, Chesnut RW, Sette A (2000). Human memory CTL response specific for influenza a virus is broad and multispecific. *Human Immunology*.

[28] Jegerlehner A, Schmitz N, Storni T, Bachmann MF (2004). Influenza A vaccine based on the extracellular domain of M2: weak protection mediated via antibody-dependent NK cell activity. *Journal of Immunology*.

[29] Mestecky J, McGhee JR (1987). Immunoglobulin A (IgA): molecular and cellular interactions involved in IgA biosynthesis and immune response. *Advances in Immunology*.

[30] Zhang G-G, Li D-X, Zhang H-H, Zeng Y-M, Chen L (2009). Enhancement of mucosal immune response against the M2eHBc+ antigen in mice with the fusion expression products of LTB and M2eHBc+ through mucosal immunization route. *Veterinary Research Communications*.

[31] Mozdzanowska K, Zharikova D, Cudic M, Otvos L, Gerhard W (2007). Roles of adjuvant and route of vaccination in antibody response and protection engendered by a synthetic matrix protein 2-based influenza A virus vaccine in the mouse. *Virology Journal*.

[32] De Filette M, Ramne A, Birkett A (2006). The universal influenza vaccine M2e-HBc administered intranasally in combination with the adjuvant CTA1-DD provides complete protection. *Vaccine*.

[33] Asahi Y, Yoshikawa T, Watanabe I (2002). Protection against influenza virus infection in polymeric Ig receptor knockout mice immunized intranasally with adjuvant-combined vaccines. *Journal of Immunology*.

[34] Renegar KB, Johnson CD, Dewitt RC (2001). Impairment of mucosal immunity by total parenteral nutrition: requirement for IgA in marine nasotracheal anti-influenza immunity. *Journal of Immunology*.

[35] Patry C, Sibille Y, Lehuen A, Monteiro RC (1996). Identification of Fc*α* receptor (CD89) isoforms generated by alternative splicing that are differentially expressed between blood monocytes and alveolar macrophages. *Journal of Immunology*.

[36] Abusugra I, Morein B (1999). Iscom is an efficient mucosal delivery system for Mycoplasma mycoides subsp. mycoides (MmmSC) antigens inducing high mucosal and systemic antibody responses. *FEMS Immunology and Medical Microbiology*.

[37] Parr EL, Parr MB (1998). Immunoglobulin G, plasma cells, and lymphocytes in the murine vagina after vaginal or parenteral immunization with attenuated herpes simplex virus type 2. *Journal of Virology*.

