# Supplementary material for: Virus-Like Particles Containing the Tetrameric Ectodomain of Influenza Matrix Protein 2 and Flagellin Induce Heterosubtypic Protection in Mice
Source: Biomed Res Int. 2013 Jul 30;2013:686549. doi: 10.1155/2013/686549 (PMC3745920; doi:10.1155/2013/686549)
Supplement: Supplementary file 1 — The supplemental material provided includes: (1) Table 1 includes the endpoint IgG titer of each group using ELISA with M2e peptides as coating antigens. (2) Table 2 includes the endpoint IgG1 titer of each group using ELISA with M2e peptides as coating antigens. (3) Table 3 includes the endpoint IgG2a titer of each group using ELISA with M2e peptides as coating antigens. (4) Table 4 includes the endpoint IgG2a titer of each group using cell surface ELISA with M2-expressing MDCK cells. (5) Table 5 includes the P values of IgG titer between different groups when using ELISA with M2e peptides as coating antigens. (6) Table 6 includes the P values of IgG1 titer between different groups when using ELISA with M2e peptides as coating antigens. (7) Table 7 includes the P values of IgG2a titer between different groups when using ELISA with M2e peptides as coating antigens. (8) Table 8 includes the P values of IgG2a titer between different groups when using cell surface ELISA with M2-expressing MDCK cells. [file 686549.f1.pdf]

Table 1.

Groups	M2e coating (IgG titre) (endpoint)					
G1: stM2e	8000.	8000.	8000.	4000.	4000.	4000.
G2: tM2e VLP	64000.	64000.	32000.	32000.	32000.	64000.
G3: tM2e/tFlic VLP	128000	128000.	128000.	64000.	64000.	64000.
G4: tM2e VLP+tFlic VLP	128000.	128000.	64000.	128000.	64000.	128000.
G5: tM2e VLP+sFlic	64000.	64000.	64000.	32000.	32000.	16000.
G6: sFlic	500.	500.	500.	500.	500.	500.

Table 2.

Groups	M2e coating (IgG1 titre) (endpoint)					
G1: stM2e	2000.	1000.	2000.	2000.	2000.	2000.
G2: tM2e VLP	64000.	64000.	32000.	32000.	32000.	32000.
G3: tM2e/tFlic VLP	64000.	128000.	128000.	64000.	64000.	64000.
G4: tM2e VLP+tFlic VLP	128000.	64000.	64000.	64000.	64000.	64000.
G5: tM2e VLP+sFlic	64000.	64000.	64000.	32000.	32000.	32000.
G6: sFlic	500.	500.	500.	500.	500.	500.

Table 3.

Groups	M2e coating (IgG2a titre) (endpoint)					
G1: stM2e	500.	1000.	1000.	1000.	1000.	1000.
G2: tM2e VLP	64000.	64000.	16000.	64000.	32000.	32000.
G3: tM2e/tFlic VLP	64000.	128000.	64000.	128000.	128000.	64000.
G4: tM2e VLP+tFlic VLP	64000.	128000.	128000.	128000.	64000.	128000.
G5: tM2e VLP+sFlic	32000.	64000.	64000.	64000.	64000.	16000.
G6: sFlic	500.	500.	500.	500.	500.	500.

Table 4.

Groups	M2 coating (IgG2a titre) (endpoint)					
G1: stM2e	1000.	2000.	2000.	2000.	2000.	4000.
G2: tM2e VLP	32000.	32000.	32000.	32000.	32000.	16000.
G3: tM2e/tFlic VLP	64000.	64000.	128000.	64000.	64000.	32000.
G4: tM2e VLP+tFlic VLP	64000.	64000.	64000.	64000.	64000.	128000.
G5: tM2e VLP+sFlic	32000.	32000.	16000.	16000.	32000.	32000.
G6: sFlic	500.	500.	500.	500.	500.	500.

Table 5.

Groups	P value (IgG) (M2e coating)(two tailed t-test)
G2 vs G3	0.0133
G2 vs G4	0.0033
G2 vs G5	0.8174

Table 6.

Groups	P value (IgG1) (M2e coating)(two tailed t-test)
G2 vs G3	0.0179
G2 vs G4	0.0296
G2 vs G5	0.5995

Table 7.

Groups	P value (IgG2a) (M2e coating)(two tailed t-test)
G2 vs G3	0.0127
G2 vs G4	0.0034
G2 vs G5	0.6732

Table 8.

Groups	P value (IgG2a) (M2 coating)(two tailed t-test)
G2 vs G3	0.0123
G2 vs G4	0.0021
G2 vs G5	0.5490
